# Prevalence and trends of active and passive smoking among Peruvian adolescents

**DOI:** 10.17843/rpmesp.2022.392.11233

**Published:** 2022-06-30

**Authors:** Antonio Bernabé-Ortiz, Rodrigo M. Carrillo-Larco

**Affiliations:** 1 CRONICAS Centro de Excelencia en Enfermedades Crónicas, Universidad Peruana Cayetano Heredia, Lima, Peru. Universidad Peruana Cayetano Heredia CRONICAS Centro de Excelencia en Enfermedades Crónicas Universidad Peruana Cayetano Heredia Lima Peru; 2 Universidad Científica del Sur, Lima, Peru. Universidad Científica del Sur Universidad Científica del Sur Lima Peru; 3 Department of Epidemiology and Biostatistics, School of Public Health, Imperial College London, London. Imperial College London Department of Epidemiology and Biostatistics School of Public Health Imperial College London London United Kingdom

**Keywords:** Tobacco, Smoking, Smoke-Free Environments, Smoking Prevention, Adolescence

## Abstract

**Objective.:**

This study aimed to assess the trends of different smoking indicators among Peruvian adolescents. Additionally, we evaluated whether such trends were different by sex or among those without previous smoking history.

**Materials and methods.:**

We analyzed the Global Youth Tobacco Survey (Global Youth Tobacco Survey 2007, 2014, 2019). Active smoking was defined according to smoking in the last 30 days. Passive smoking was assessed based on exposure to tobacco inside and outside the household, both overall and daily. Analyses considered the sample design.

**Results.:**

A total of 17,047 records (9,869 in 2007, 3,424 in 2014, and 3,754 in 2019) were analyzed; the mean age was 14 years, and 49.9% were women. Previous smoking history was reported in 26.6% of the records; such prevalence fell from 45.2% (2007), to 25.3% (2014), and to 19.4% (2019, p-value for trend < 0.001), whereas active smoking fell from 17.1% (2007) to 8.7% (2014) and to 5.7% (2019). The overall prevalence of passive smoking inside the household fell from 24.7% to 12.9% and 10.4% (p-value <0.001), whereas the overall prevalence of passive smoking outside the household dropped from 46.3% to 39.4% and 36.3% (p-value <0.001) during the same period. The reduction of the smoking indicators was observed mainly among women than in men.

**Conclusion.:**

There is evidence of a sustained reduction in smoking indicators in Peruvian adolescents. Passive smoking outside the household continues to be common, calling for strengthening current tobacco control policies.

## INTRODUCTION

Smoking, in any form, was the cause of 8.7 million deaths in 2019, and responsible for 15.4% of all deaths that year ^1^. Overall, many countries, especially those with high economic incomes, have seen a considerable reduction in smoking prevalence in both males and females, but these changes have not occurred in poorer countries ^2^.

Exposure to tobacco (active or passive) during adolescence still continues to be a public health problem worldwide, due to the known harmful effect it can have on health ^3^ ^,^ ^4^. Although the prevalence of smoking during adolescence has decreased in most countries, it has changed little in other countries over the past two decades ^5^. Moreover, one study reported that approximately 12.5% of adolescents who had never smoked were susceptible to smoking, and this susceptibility was highest in the Americas region ^6^.

In Latin American countries, several regulations and laws have been created to reduce tobacco consumption and exposure. In Peru, the General Law for the Prevention and Control of the Risks of Tobacco Consumption (Law 28705, known as the anti-tobacco law) was enacted in 2006 and establishes that the minimum age for tobacco consumption is 18 years, prohibiting its consumption in establishments dedicated to health or education ^7^. A subsequent amendment to this law ^8^ prohibited smoking in enclosed places and in any means of public transportation and established the obligation to post signs in enclosed spaces prohibiting smoking. Despite the implementation of the law and its subsequent modification, which occurred several years ago, few studies have evaluated changes in smoking patterns in adolescents. A recent study, using a quasi-experimental design, found that the anti-smoking law had almost negligible effects on birth weight and the incidence of prematurity at the population level ^9^.

Consequently, this study aimed to evaluate the trends in different indicators of smoking among Peruvian adolescents using surveys with standardized methodology. Likewise, we evaluated trends in these indicators according to groups of interest (by sex and in those with no previous smoking history).

**KEY MESSAGES**

Motivation for the study: Despite the different laws and modifications that have been implemented regarding tobacco consumption, there is no evaluation of smoking trends in Peruvian adolescents.

Main findings: The results show a sustained decrease in different indicators of smoking among Peruvian adolescents. However, passive smoking outside the home continues to be prevalent.

Implications: The findings suggest the need to strengthen current tobacco control policies in the country to particularly reduce passive smoking in adolescents.

## MATERIALS AND METHODS

### Study design

In this study we analyzed three population-based surveys based on the Global Youth Tobacco Survey (GYTS), a group of different studies conducted to monitor adolescent tobacco use and guide the implementation and evaluation of tobacco prevention and control programs ^10^. For the analysis we used information from surveys conducted in Peru during 2007, 2014 and 2019 ^11^.

### Characteristics of the GYTS

The GYTS is a cross-sectional, self-administered, nationally representative survey that focuses primarily on tobacco use and related factors in school adolescents aged 12-16 years worldwide ^12^. The GYTS uses a standard methodology to construct the sampling frame, select schools and classrooms, prepare and administer questionnaires, follow consistent field procedures, and use consistent data management procedures for data processing and analysis. The World Health Organization (WHO) provides technical support to all participating countries ^10^.

The GYTS applies a two-stage sampling strategy to select a random, nationally representative sample and its methodology has been detailed above ^12^. Briefly, in the first phase, schools are selected randomly and proportional to enrollment size. In the second phase, classrooms within the selected schools are randomly selected. All schoolchildren within the selected classrooms are eligible to voluntarily participate in the survey. The standardized questionnaire is translated from English into the local language by researchers in each country, and then translated back into the original language to ensure accuracy. The GYTS research protocol is approved by WHO and the U.S. Centers for Disease Control and Prevention (CDC) ^13^.

### Definition of variables

The main variables were active smoking, passive smoking at home, and passive smoking outside the home, and were based on GYTS questions available in the three different questionnaires evaluated (2007, 2014, and 2019), and which have been used in other similar studies ^5^ ^,^ ^14^ ^,^ ^15^.

The question “Have you ever tried or experimented with cigarettes, even one or two puffs?” served to generate the variable prior smoking history. Those who answered “No” were classified as having never smoked, while those who answered "Yes" were considered to have a history of prior exposure. The latter group was further asked “How old were you when you first tried a cigarette?”. The response options for this last question were: I have never tried cigarettes, before the age of 7 years, between 8-9 years, between 10-11 years, between 12-13 years, between 14-15 years and at the age of 16 years. For descriptive purposes, this variable was recategorized into <10 years, 10-13 years, and 14-16 years.

The question, “During the past 30 days, how many days did you smoke cigarettes?” was used to define active smoking. The response options were: no days, 1 or 2 days, 3 to 5 days, 6 to 9 days, 10 to 19 days, 20 to 29 days, and every day. For analysis purposes, the options were collapsed into two categories: “No use” compatible with no days, and “Some use” if at least 1 day of tobacco use in the past 30 days was reported.

Passive smoking at the home was assessed by the question, “During the last 7 days, how many days has someone smoked inside your home, in your presence?”. The response options were 0 days, 1-2 days, 3-4 days, 5-6 days, and 7 days. For analysis purposes, this question was categorized in two different ways to generate two different variables: passive smoking at home, defined as any exposure to tobacco, passively, inside the home, that is, those who reported at least 1 day of exposure in the last week; while the second variable was daily passive smoking at home, defined as passive and continuous exposure to tobacco, that is, during all 7 days of the previous week.

Similarly, passive smoking outside the home was also assessed, using different questions. In 2007, we used the question “During the last 7 days, how many days have people smoked in your presence, in places other than your home?”; however, in 2014 and 2019, two questions were used to capture this information: “During the last 7 days, how many days has someone smoked in your presence, inside an enclosed public place, other than your home?” and “During the last 7 days, how many days has someone smoked in your presence, in any outdoor public place?”. For purposes of comparison across years, these last two questions were merged, with the sum of the two being considered the total out-of-home exposure. The response options for all these questions were 0 days, 1-2 days, 3-4 days, 5-6 days, and 7 days. For analysis purposes, two different variables were generated: passive smoking outside the home, defined as any exposure to tobacco, passively, outside the home (i.e., those who reported ≥1 day of exposure in the last week); while the second variable was daily passive smoking outside the home, i.e., during all 7 days of the previous week.

Other covariates used for descriptive purposes and for subgroup analysis were: sex (male vs. female), age (12-14 vs. 15-16 years), education level (high school grades 1-5), and year of study (2007, 2014, and 2019).

### Statistical analysis

All analyses were performed considering the two-stage design of each survey using the denormalized weighting of each survey individually and considering the sampling design and nonresponse rates. Missing values were not considered for the estimation of point estimates (e.g., prevalences); however, they were included for the estimation of standard errors and hence 95% confidence intervals (95% CI) using the “subpop” command in STATA as previously reported ^16^. Analyses by subgroups of interest (sex and those with no previous smoking history) were carried out using the appropriate option for subpopulation management.

Initially, the population was described according to the year of study (2007, 2014, and 2019) and the profile of participants was compared using the chi-square test with Rao and Scott’s second-order correction for categorical variables ^17^. Then, we estimated the prevalence of the variables of interest and the respective 95% CIs. These estimates were calculated by year of study and globally. We evaluated the trend of the chosen smoking indicators over time using the trend score test and using the year 2007 as the reference category. STATA 16 for Windows (StataCorp, College Station, TX, USA) was used for statistical analysis and a p < 0.05 was considered statistically significant.

### Ethics

The survey data are freely available without personal identifiers, and because of this, ethical review was not considered indispensable for the present work.

## RESULTS

### Description of the study population

A total of 19,551 records from male and female students were collected in the GYTS (11,585 in 2007, 3818 in 2014, and 4148 in 2019). Of these, 2504 (12.8%) were excluded due to incomplete data on the variables of interest (sex, smoking history, and passive smoking). Thus, 17047 records (9869 in 2007, 3424 in 2014, and 3754 in 2019) were included in the analyses, mean age 14.1 (SD: 1.3) years, and 49.9% female. The distribution of the study population according to sex, age groups, and year of study did not vary between study years (Table 1).

**Table 1:** Description of the study population by study year: 2007, 2014, and 2019 Global Youth Smoking Survey.

Variables	Study year			p-value ^a^
	2007	2014	2019	
	(N = 9869)	(N = 3424)	(N = 3754)	
	n (%)	n (%)	n (%)	
Sex				0.993
Female	4737 (49.5)	1773 (49.9)	1926 (50.0)	
Male	5132 (50.5)	1651 (50.1)	1828 (50.0)	
Age				0.564
12 - 14 years	6055 (61.3)	2121 (59.1)	2285 (61.2)	
15 - 16 years	3814 (38.7)	1303 (40.9)	1469 (38.8)	
Education level				0.985
1.° Secondary school	2534 (24.1)	844 (23.4)	844 (22.6)	
2.° Secondary school	2175 (24.4)	751 (23.2)	871 (24.0)	
3.° Secondary school	2335 (21.7)	797 (21.4)	814 (21.4)	
4.° Secondary school	1675 (19.1)	660 (19.1)	732 (18.2)	
5.° Secondary school	1131 (10.7)	352 (12.9)	479 (13.8)	
History of tobacco use				<0.001
No	5490 (54.8)	2497 (74.7)	3008 (80.6)	
Yes	4379 (45.2)	927 (25.4)	746 (19.4)	
Age at first smoking ^b^				0.045
<10 years	414 (9.6)	108 (14.0)	62 (9.8)	
10 - 13 years	2208 (53.4)	423 (47.5)	330 (46.4)	
14 - 16 years	1551 (37.0)	328 (38.6)	302 (43.8)	
Active smoking				<0.001
No	7830 (82.9)	2938 (91.4)	3450 (94.3)	
Yes	1517 (17.1)	332 (8.7)	206 (5.7)	

All estimates were calculated considering the study design.

a p-value calculated using the chi-square homogeneity test.

b Estimated on those who reported having history of tobacco use.

### Prevalence and trends of active smoking

The overall prevalence of smoking history was 26.6% (95% CI: 24.7% - 28.4%); however, that estimate dropped from 45.2% (95% CI: 42.3% - 48.1%) in 2007 to 25.3% (95% CI: 22.1% - 28.6%) in 2014, and then to 19.4% (95% CI: 16.3% - 22.5%) in 2019 (p-value for trends < 0.001). Despite an apparent delay in the age of smoking initiation, there was no significant difference in that variable over time (Table 1).

On the other hand, the prevalence of active smoking was 8.9% (95% CI: 7.7% - 10.1%), with a drop from 17.1% (95% CI: 14.9% - 19.4%) in 2007 to 8.6% (95% CI: 6.1% - 11.2%) in 2014, and then to 5.7% (95% CI: 4.5% - 7.0%) in 2019 (p-value for trends < 0.001).

Although the decrease in smoking history prevalence was significant in both sexes, the decline was greater in females (from 50.7% in 2007 to 16.7% in 2019) compared to males (39.9% in 2007 to 22.1% in 2019). Similar findings were seen in active smoking (Table 2).

**Table 2:** Trend over time in prevalence of active and passive smoking according to groups of interest: 2007, 2014 and 2019 Global Youth Smoking Survey.

Variables	Study year			p-value ^a^
	2007	2014	2019	
	% (95% CI)	% (95% CI)	% (95% CI)	
Women				
History of previous smoking	50.7 (46.9 - 54.5)	20.1 (16.1 - 24.0)	16.7 (13.4 - 19.9)	<0.001
Active smoking	21.2 (18.6 - 23.8)	6.5 (4.2 - 8.8)	4.4 (3.0 - 5.8)	<0.001
Passive smoking at home	24.6 (21.5 - 27.7)	12.9 (10.3 - 15.5)	10.8 (9.3 - 12.3)	<0.001
Daily passive smoking at home	4.0 (2.8 - 5.1)	1.6 (0.8 - 2.4)	1.5 (0.9 - 2.1)	<0.001
Passive smoking outside the home	47.0 (43.7 - 50.4)	40.5 (36.9 - 44.1)	39.2 (34.9 - 43.6)	0.007
Daily passive smoking outside the home	6.2 (5.0 - 7.5)	4,1 (2.9 - 5.3)	3.9 (3.0 - 4.8)	0.006
Men				
History of previous smoking	39.9 (36.7- 43.0)	30.6 (26.4 - 34.8)	22.1 (17.8 - 26.5)	<0.001
Active smoking	13.1 (10.5 - 15.7)	10.9 (7.6 - 14.1)	7.0 (5.2 - 8.8)	<0.001
Passive smoking at home	24.8 (21.5 - 28.1)	12.9 (10.6 - 15.1)	9.9 (7.6 - 12.3)	<0.001
Daily passive smoking at home	2.7 (1.8 - 3.5)	1.9 (1.0 - 2.9)	1.4 (0.7 - 2.1)	0.108
Passive smoking outside the home	45.7 (41.6 - 49.8)	38.3 (33.8 - 42.8)	33.4 (28.6 - 38.1)	<0.001
Daily passive smoking outside the home	5.6 (4.7 - 6.5)	5.9 (4.5 - 7.3)	4.2 (3.2 - 5.2)	0.093
Those who have never smoked				
Passive smoking at home	20.1 (17.3 - 23.0)	8.2 (7.1 - 9.3)	7.7 (6.4 - 9.0)	<0.001
Daily passive smoking at home	2.1 (1.4 - 2.8)	1.0 (0.5 - 1.4)	0.7 (0.4 - 1.0)	<0.001
Passive smoking outside the home	38.0 (34.3 - 41.8)	32.5 (29.2 - 35.8)	31.0 (26.7 - 35.2)	0.026
Daily passive smoking outside the home	4.2 (3.4 - 5.0)	3.6 (2.8 - 4.3)	2.9 (2.0 - 3.7)	0.101

All estimates were calculated considering the study design.

a p-value calculated using trend tests considering the study design.

### Prevalence and trend of passive smoking

**Figure 1:**
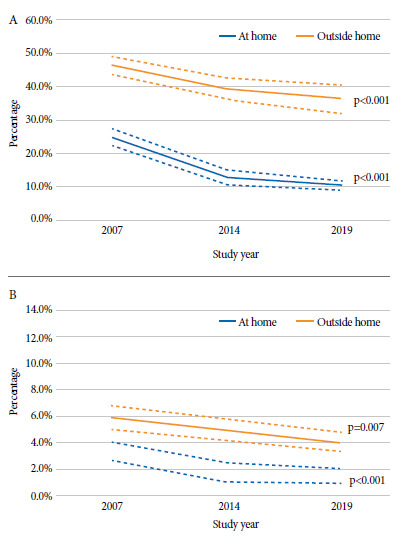
Trend over time in the prevalence of (A) passive smoking and (B) daily passive smoking in adolescents in Peru: Global Youth Tobacco Survey 2007, 2014 and 2019.

The prevalence of passive smoking inside the home was 14.0% (95% CI: 12.9% - 15.2%). That estimate decreased from 24.7% (95% CI: 22.0% - 27.3%) in 2007 to 12.9% (95% CI: 10.6% - 15.1%) in 2014, subsequently falling to 10.4% (95% CI: 9.0% - 11.7%) in 2019 (p-trend value < 0.001). On the other hand, the overall prevalence of passive smoking outside the home was 39.4% (95% CI: 37.2% - 41.6%), with a drop in estimates from 46.3% (95% CI: 43.7% - 49.0%) in 2007 to 39.4% (95% CI: 36.0% - 42.8%) in 2014, and to 36.3% (95% CI: 32.1% - 40.5%) in 2019 (p-value of trends < 0.001) (Figure 1A).

Similarly, the prevalence of daily passive smoking inside the home was 1.9% (95% CI: 1.5% - 2.3%), with a drop from 3.3% (95% CI: 2.6% - 4.0%) in 2007 to 1.8% (95% CI: 1.1% - 2.5%) in 2014, and then to 1.5% (95% CI: 0.9% - 2.0%) in 2019 (p-value for trends < 0.001). On the other hand, the overall prevalence of daily passive smoking outside the home was 4.7% (95% CI: 4.2% - 5.3%), and similar to previous estimates, there was a drop over time from 5.9% (95% CI: 5.1% - 6.7%) in 2007 to 5.0% (95% CI: 4.0% - 5.9%) in 2014, and then to 4.0% (95% CI: 3.3% - 4.8%) in 2019 (p-trend p-value = 0.007) (Figure 1B).

### Active and passive smoking by groups of interest

Table 2 shows the estimates and trends of the different smoking markers selected according to sex and in those without a history of smoking. In the case of women, all markers decreased over time, but this was not the case for daily passive smoking at home (p-value of trends = 0.108) and daily passive smoking outside the home (p-value of trends = 0.093) in men.

In the group with no history of previous smoking (i.e., those who reported never having smoked), all indicators decreased during the study period; however, although the prevalence of daily passive smoking outside the home declined during the study period, the difference was not significant (p-value for trends = 0.101) (Table 2).

## DISCUSSION

According to our results, since 2007, several indicators of smoking among Peruvian adolescents have decreased, although it is greater in females than in males. Despite the fact that by 2019, only 1 in 5 adolescents between 12 and 16 years of age reported having a history of smoking, this is far from the target for tobacco use in children under 18 years of age. Likewise, the prevalence of passive smoking, especially outside the home, remains high, which may be more relevant in those with no previous smoking history.

Active tobacco use is a preventable risk factor for morbidity and mortality worldwide. Despite the reduction in tobacco use over time, the prevalence of prior smoking history and current tobacco use (in the past 30 days) remains high. However, our 2019 prevalence estimates are lower than those of a global analysis of smoking prevalence in adolescents aged 13 to 15 years ^5^: 6.1% in females and 11.3% in males (vs. 4.4% and 7.0%, respectively, in our study), and lower than those of other countries in the region ^18^ ^,^ ^19^.

Similarly, passive smoking exposure is also preventable and, despite depending on the reduction of active smoking, its effects are relevant because it increases the likelihood of an adolescent becoming a smoker, as well as increasing susceptibility to smoking ^20^. The prevalence of passive smoking reported here is much lower than that reported in other studies globally ^14^, both inside and outside the home ^15^. However, the prevalence of passive smoking remains high, especially outside the home.

Peru signed and ratified the Framework Convention on Tobacco Control (FCTC) developed by the WHO in 2004. This framework includes a total ban on advertising, promotion and sponsorship of tobacco products; strong health warnings on cigarette packaging; protection from exposure to tobacco smoke in workplaces and public places, as well as in public transport; and measures to reduce illicit tobacco trade ^21^. These initiatives, and their subsequent strengthening, could explain the reduction observed in the various indicators.

The observed changes in the prevalence of different indicators of adolescent smoking suggest that tobacco control policies have improved in Peru, especially those related to protection from tobacco smoke in the home, in public places, and in transportation ^22^. However, our results suggest that policies on tobacco use should be strengthened, especially outside the home.

According to a previous study that used data from the 2007 GYTS in Peru and compared it with other countries in the region, and despite a marked reduction in adolescent tobacco use, Peru was rated by WHO as one of the countries with the lowest implementation of anti-smoking policies ^23^. Even in that report, Peru was the only country where cities had a high exposure to passive smoking.

The enactment of Law 29517 (2011) that banned smoking in public places in a much more restrictive manner than the previous law ^8^ may have helped in the subsequent reduction in exposure to passive smoking. Thus, protection of adolescents from existing forms of smoking, especially passive smoking at home and in public places, should be critical in reducing smoking initiation in this age group ^24^. According to our results, it is necessary, then, to continue strengthening existing policies to achieve a greater reduction in the indicators of active and passive smoking.

This researched used GYTS data at different times in Peru. Additionally, it used representative samples with standardized methodology and questions. The results could have important implications for the generation and evaluation of policies to control tobacco use in Peruvian adolescents. However, this study has some limitations that deserve discussion. First, the GYTS uses a self-report instrument to determine information on tobacco use and, therefore, there could be recall or social desirability biases that could affect the results. Second, only those students who were present during the conduct of the survey were assessed and, thus, may affect the generalizability of the results. Third, although the 2014 and 2019 surveys are nationally representative, the 2007 survey was conducted only in some of Peru’s large cities (Huancayo, Ica, Lima, Trujillo and Tarapoto) ^25^, thus showing a larger decrease than what might actually have occurred. In spite of this, our results show a clear decreasing trend in the obtained smoking indicators and are comparable with what has been observed in other studies ^5^ ^,^ ^14^ ^,^ ^15^. Fourth, indicators of passive smoking outside the home were assessed using a single question in the 2007 survey, and two questions in the 2014 and 2019 surveys, which could affect the results. However, the estimates appear to be consistent with other studies ^23^. Finally, certain variables such as socioeconomic status were not collected as part of the survey. This factor could be of relevance given the high cost of tobacco products, which could be useful to better characterize the results shown.

In conclusion, our results show a sustained reduction in several indicators of smoking in Peruvian adolescents between 12 and 16 years of age, being higher in females than in males. However, the prevalence of passive smoking outside the home remains high, which may require a strengthening of current tobacco control policies in the country.
