# Supplementary material for: Prevalence and trends of active and passive smoking among Peruvian adolescents
Source: Rev Peru Med Exp Salud Publica. 2022 Jun 30;39(2):193–200. doi: 10.17843/rpmesp.2022.392.11233 (PMC11397579; doi:10.17843/rpmesp.2022.392.11233)
Supplement: Translation

# Translation: Prevalencia y tendencias de tabaquismo activo y pasivo en adolescentes peruanos

**Authors:** Antonio Bernabé-Ortiz, Rodrigo M. Carrillo-Larco

**Affiliations:** 1 CRONICAS Centro de Excelencia en Enfermedades Crónicas, Universidad Peruana Cayetano Heredia, Lima, Perú.; 2 Universidad Científica del Sur, Lima, Perú.; 3 Department of Epidemiology and Biostatistics, School of Public Health, Imperial College London, London.

**Keywords:** Tabaquismo, Fumar, Ambientes Libres de Humo, Prevención del Hábito de Fumar, Adolescencia

## Abstract

**Objetivo.:**

El objetivo del presente estudio fue evaluar las tendencias ocurridas en diferentes indicadores de tabaquismo en adolescentes peruanos. Asimismo, se evaluó las tendencias en dichos indicadores de acuerdo a sexo y en aquellos sin antecedente de tabaquismo previo.

**Materiales y métodos.:**

El presente estudio usa la Encuesta Mundial sobre Tabaquismo en Jóvenes (Global Youth Tobacco Survey 2007, 2014, 2019). Tabaquismo activo se definió según el consumo de tabaco en los últimos 30 días. Tabaquismo pasivo se definió en función a exposición a tabaco dentro y fuera del hogar, tanto global como en forma diaria. Los análisis consideraron el diseño muestral.

**Resultados.:**

Un total de 17 047 registros (9869 en el 2007, 3424 en el 2014, y 3754 en el 2019) fueron analizados; edad media 14 años y 49,9% mujeres. El 26,6% reportó antecedente de tabaquismo previo; dicha prevalencia cayó de 45,2% (2007), a 25,3% (2014), y a 19,4% (2019, p de tendencias < 0,001), mientras que el tabaquismo activo cayó de 17,1% (2007) a 8,7% (2014) y a 5,7% (2019). La prevalencia global de tabaquismo pasivo en el hogar cayó de 24,7%, a 12,9% y a 10,4% (p de tendencias <0,001), mientras que la prevalencia global de tabaquismo pasivo fuera del hogar se redujo de 46,3%, a 39,4%, y a 36,3% (p < 0,001) en ese lapso. La caída en los indicadores de tabaquismo se vio principalmente en mujeres que en varones.

**Conclusión.:**

Se evidencia una reducción sostenida en los indicadores de tabaquismo en adolescentes peruanos. El tabaquismo pasivo fuera del hogar continúa siendo frecuente requiriendo el fortalecimiento de las actuales políticas de control de tabaco.

## INTRODUCCIÓN

El tabaquismo, en cualquiera de sus formas, fue causa de 8,7 millones de muertes en el 2019, y responsable de 15,4% de todas las muertes ocurridas ese año ^1^. En general, muchos países, especialmente aquellos de altos ingresos económicos, han visto una reducción considerable en la prevalencia de tabaquismo tanto en varones como en mujeres, pero estos cambios no se han dado en los países más pobres ^2^.

En la adolescencia, la exposición al tabaco (activa o pasiva) todavía continúa siendo un problema de salud pública en todo el mundo, debido al conocido efecto deletéreo que puede tener sobre la salud ^3^ ^,^ ^4^. Aunque la prevalencia de tabaquismo durante la adolescencia ha disminuido en la mayoría de países, dicha prevalencia ha variado muy poco en otros países en las últimas dos décadas ^5^. Más aun, un estudio reportó que aproximadamente el 12,5% de los adolescentes que nunca habían fumado eran susceptibles a fumar, y esta susceptibilidad fue mayor en la región de las Américas ^6^.

En los países de Latinoamérica se han creado diversas normas y leyes para reducir el consumo y exposición al tabaco. En el Perú, la Ley General para la Prevención y Control de los Riesgos del Consumo del Tabaco (Ley 28705, conocida como la ley antitabaco) fue promulgada en el 2006 y establece en su contenido que la edad mínima para el consumo de tabaco es de 18 años, prohibiendo su consumo en establecimientos dedicados a la salud o la educación ^7^. Una modificación posterior a dicha ley ^8^ prohibía fumar en lugares cerrados y en cualquier medio de transporte público y establecía la obligatoriedad de anuncios en espacios cerrados prohibiendo fumar. A pesar de que la implementación de la ley, y su posterior modificación, ocurrió hace varios años, pocos estudios han evaluado los cambios en los patrones de tabaquismo en adolescentes. Así, un estudio reciente, usando un diseño cuasiexperimental, encontró que la ley antitabaco tuvo efectos casi negligibles en el peso al nacer y en la incidencia de prematuridad a nivel poblacional ^9^. 

Como consecuencia, el objetivo principal del presente estudio fue evaluar las tendencias ocurridas en diferentes indicadores de tabaquismo en adolescentes peruanos usando encuestas con metodología estandarizada. Asimismo, se evaluó las tendencias en dichos indicadores de acuerdo a grupos de interés (por sexo y en aquellos sin antecedente de tabaquismo previo).

**MENSAJE CLAVE**

Motivación para realizar el estudio: a pesar de las diferentes leyes y modificaciones que se han dado sobre el consumo de tabaco, no existe una evaluación de las tendencias de tabaquismo en adolescentes peruanos.

Principales hallazgos: los resultados muestran una reducción sostenida en diferentes indicadores de tabaquismo en adolescentes peruanos. Sin embargo, el tabaquismo pasivo fuera del hogar continúa siendo frecuente.

Implicancias: los hallazgos sugieren la necesidad de fortalecer las actuales políticas de control de tabaco en el país para reducir sobre todo el tabaquismo pasivo en adolescentes.

## MATERIALES Y MÉTODOS

### Diseño del estudio

Para el presente trabajo se analizaron tres encuestas poblacionales basados en la Encuesta Mundial sobre Tabaquismo en Jóvenes (Global Youth Tobacco Survey o GYTS, de sus siglas inglés), un grupo de diferentes estudios realizados para monitorear el consumo de tabaco en adolescentes y guiar la implementación y evaluación de los programas de prevención y control de tabaquismo ^10^. Para el análisis se usó la información correspondiente al Perú de las encuestas realizadas en el 2007, 2014 y 2019 ^11^.

### Características del GYTS

El GYTS es una encuesta transversal, autoadministrada y representativa a nivel nacional, que se centra principalmente en el consumo de tabaco y los factores relacionados en adolescentes escolares de 12 a 16 años de todo el mundo ^12^. El GYTS utiliza una metodología estándar para construir el marco muestral, seleccionar los colegios y salones de clase, preparar y administrar cuestionarios, seguir procedimientos de campo consistentes, y usar procedimientos de manejo de datos consistentes para el procesamiento y análisis de datos. La Organización Mundial de la Salud (OMS) da soporte técnico a todos los países participantes ^10^.

El GYTS aplica una estrategia de muestreo bietápico para seleccionar una muestra aleatoria, que sea representativa a nivel nacional y su metodología ha sido detallada anteriormente ^12^. Brevemente, en la primera fase, las escuelas se seleccionan aleatoriamente proporcional al tamaño de la matrícula. En la segunda fase, los salones dentro de las escuelas seleccionadas se eligen en forma aleatoria. Todos los escolares dentro de los salones seleccionados son elegibles para participar voluntariamente en la encuesta. El cuestionario estandarizado es traducido del inglés al idioma local por investigadores en cada país, y luego traducido al idioma original para garantizar su precisión. El protocolo de investigación del GYTS está aprobado por la OMS y los Centros para el Control y la Prevención de Enfermedades (CDC) de los EE. UU. ^13^.

### Definición de variables

Las variables principales fueron tabaquismo activo, tabaquismo pasivo en el hogar y tabaquismo pasivo fuera del hogar, y estuvieron basadas en preguntas del GYTS disponibles en los tres diferentes cuestionarios evaluados (2007, 2014, y 2019), y que han sido usadas en otros estudios similares ^5^ ^,^ ^14^ ^,^ ^15^.

La pregunta «¿Alguna vez ha probado o experimentado con cigarrillos, aunque sea uno o dos pitadas?» sirvió para generar la variable antecedente de tabaquismo previo. Aquellos que respondieron «No» fueron clasificados como que nunca habían fumado, mientras que los que respondieron «Sí» fueron considerados como que tenían antecedente de exposición previa. A este último grupo además se le preguntó «¿Qué edad tenías cuando probaste un cigarrillo por primera vez?». Las opciones de respuesta de esta última pregunta fueron: Nunca he probado cigarrillos, antes de los 7 años inclusive, entre 8-9 años, entre 10-11 años, entre 12-13 años, entre 14-15 años y a los 16 años. Para fines descriptivos, esta variable fue recategorizada en <10 años, 10-13 años, y de 14-16 años. 

La pregunta: «Durante los últimos 30 días, ¿cuántos días fumó cigarrillos?» se utilizó para definir tabaquismo activo. Las opciones de respuesta fueron: Ningún día, 1 o 2 días, 3 a 5 días, 6 a 9 días, 10 a 19 días, 20 a 29 días, y todos los días. Para fines de análisis se colapsaron las opciones en dos categorías: «Ningún consumo» compatible con ningún día, y «Algún consumo» si reportó al menos 1 día de consumo de tabaco en los últimos 30 días. 

El tabaquismo pasivo en el hogar fue evaluado mediante la pregunta: «Durante los últimos 7 días, ¿cuántos días ha fumado alguien dentro de tu casa, en tu presencia?». Las opciones de respuesta fueron 0 días, 1-2 días, 3-4 días, 5-6 días, y 7 días. Para fines de análisis, esta pregunta se categorizó de dos formas distintas para generar dos variables diferentes: tabaquismo pasivo en el hogar, definido como cualquier exposición al tabaco, en forma pasiva, dentro del hogar, es decir, aquellos que reportaron al menos 1 día de exposición en la última semana; mientras que la segunda variable fue tabaquismo pasivo diario en el hogar, definido como la exposición continua al tabaco en forma pasiva, es decir, durante todos los 7 días de la semana previa evaluada.

De la misma forma, también se evaluó el tabaquismo pasivo fuera del hogar, mediante diferentes preguntas. En el 2007 se usó la pregunta «Durante los últimos 7 días, ¿cuántos días han fumado personas en tu presencia, en lugares distintos a su casa?»; sin embargo, en el 2014 y 2019 se usaron dos preguntas para capturar esta información: «Durante los últimos 7 días, ¿cuántos días ha fumado alguien en tu presencia, dentro de un lugar público cerrado, que no sea tu hogar?» y «Durante los últimos 7 días, ¿cuántos días ha fumado alguien en tu presencia, en cualquier lugar público al aire libre?». Para fines de comparación entre años, estas dos últimas preguntas fueron unidas, considerándose la suma de ambas como el total de exposición fuera del hogar. Las opciones de respuesta de todas estas preguntas fueron 0 días, 1-2 días, 3-4 días, 5-6 días, y 7 días. Para fines de análisis, se generó dos variables diferentes: tabaquismo pasivo fuera del hogar, definido como cualquier exposición al tabaco, en forma pasiva, fuera del hogar (es decir, aquellos que reportaron ≥1 día de exposición en la última semana); mientras que la segunda variable fue tabaquismo pasivo diario fuera del hogar, es decir, durante todos los 7 días de la semana previa evaluada.

Otras covariables que se usaron con fines de descripción y para el análisis por subgrupos fueron: sexo (varón vs. mujer), edad (12-14 vs. 15-16 años), nivel de educación (secundaria de 1.° a 5.° grado), y año de estudio (2007, 2014, y 2019).

### Análisis estadístico

Todos los análisis fueron realizados considerando el diseño bietápico de cada encuesta utilizando la ponderación desnormalizada de cada encuesta en forma individual y considerando el diseño de muestreo y las tasas de no respuesta. Los valores faltantes no fueron considerados para la estimación de los estimados puntuales (ej., prevalencias); sin embargo, sí fueron incluidos para la estimación de los errores estándar y por ende de los intervalos de confianza al 95% (IC 95%) usando el comando «subpop» en STATA como ha sido reportado anteriormente ^16^. Los análisis por subgrupos de interés (sexo y aquellos que no tenían antecedente de tabaquismo previo) se realizaron utilizando la opción apropiada para manejo de subpoblaciones. 

Inicialmente, se describió a la población de acuerdo al año de estudio (2007, 2014, y 2019) y se comparó el perfil de participantes usando la prueba de chi cuadrado con la corrección de segundo orden de Rao y Scott para variables categóricas ^17^. Luego, se estimó la prevalencia de las variables de interés y los IC 95% respectivos. Dichas estimaciones se realizaron de forma global y por año de estudio. La tendencia en el tiempo de los indicadores de tabaquismo escogidos fue evaluada usando la prueba de puntuación para tendencias usando el año 2007 como categoría de referencia. Para el análisis estadístico se utilizó STATA 16 para Windows (StataCorp, College Station, TX, EE.UU.) y un p < 0,05 se consideró estadísticamente significativo.

### Ética

Los datos de las encuestas están disponibles libremente sin identificadores personales, y debido a eso, la revisión ética no fue considerada indispensable para el presente trabajo.

## RESULTADOS

### Descripción de la población de estudio

Un total de 19 551 registros provenientes de estudiantes de ambos sexos, se colectaron en el GYTS (11 585 en el 2007, 3818 en el 2014, y 4148 en el 2019). De ellos, 2504 (12,8%) fueron excluidos por datos incompletos en las variables de interés (sexo, historia de tabaquismo y tabaquismo pasivo). Así, 17047 registros (9869 en el 2007, 3424 en el 2014, y 3754 en el 2019) se incluyeron en los análisis, edad media de 14,1 (DE: 1,3) años, y 49,9% mujeres. La distribución de la población de estudio de acuerdo a sexo, grupos de edad y año de estudio no varió entre los años de estudio (Tabla 1).

**Tabla 1:** Descripción de la población de estudio por año de estudio: Encuesta Mundial sobre Tabaquismo en Jóvenes 2007, 2014 y 2019.

Variables	Año de estudio			Valor de p ^a^
	2007	2014	2019	
	(N = 9869)	(N = 3424)	(N = 3754)	
	n (%)	n (%)	n (%)	
Sexo				0,993
Mujeres	4737 (49,5)	1773 (49,9)	1926 (50,0)	
Varones	5132 (50,5)	1651 (50,1)	1828 (50,0)	
Edad				0,564
12 - 14 años	6055 (61,3)	2121 (59,1)	2285 (61,2)	
15 - 16 años	3814 (38,7)	1303 (40,9)	1469 (38,8)	
Nivel de educación				0,985
1.° Secundaria	2534 (24,1)	844 (23,4)	844 (22,6)	
2.° Secundaria	2175 (24,4)	751 (23,2)	871 (24,0)	
3.° Secundaria	2335 (21,7)	797 (21,4)	814 (21,4)	
4.° Secundaria	1675 (19,1)	660 (19,1)	732 (18,2)	
5.° Secundaria	1131 (10,7)	352 (12,9)	479 (13,8)	
Antecedente de consumo de tabaco				<0,001
No	5490 (54,8)	2497 (74,7)	3008 (80,6)	
Sí	4379 (45,2)	927 (25,4)	746 (19,4)	
Edad a la que fumó por primera vez ^b^				0,045
<10 años	414 (9,6)	108 (14,0)	62 (9,8)	
10 - 13 años	2208 (53,4)	423 (47,5)	330 (46,4)	
14 - 16 años	1551 (37,0)	328 (38,6)	302 (43,8)	
Tabaquismo activo				<0,001
No	7830 (82,9)	2938 (91,4)	3450 (94,3)	
Sí	1517 (17,1)	332 (8,7)	206 (5,7)	

Todas las estimaciones fueron hechas tomando en cuenta el diseño del estudio.

a Valor de p calculado usando la prueba de homogeneidad de chi cuadrado.

b Estimado sobre aquellos que reportaron tener historia de consumo de tabaco.

### Prevalencia y tendencias de tabaquismo activo

La prevalencia global de antecedente de tabaquismo fue de 26,6% (IC 95%: 24,7% - 28,4%); sin embargo, dicho estimado cayó de 45,2% (IC 95%: 42,3% - 48,1%) en el 2007 a 25,3% (IC 95%: 22,1% - 28,6%) en el 2014, y luego a 19,4% (IC 95%: 16,3% - 22,5%) en el 2019 (p-valor de tendencias < 0,001). A pesar de un aparente retraso en la edad de inicio de consumo de tabaco, no hubo diferencia significativa en dicha variable en el tiempo (Tabla 1).

De otro lado, la prevalencia de tabaquismo activo fue de 8,9% (IC 95%: 7,7% - 10,1%), con una caída de 17,1% (IC 95%: 14,9% - 19,4%) en el 2007 a 8,6% (IC 95%: 6,1% - 11,2%) en el 2014, y luego a 5,7% (IC 95%: 4,5% - 7,0%) en el 2019 (p-valor de tendencias < 0,001). 

Aunque la reducción en la prevalencia de antecedente de tabaquismo se dio en forma significativa en ambos sexos, la caída fue mayor en mujeres (de 50,7% en el 2007 a 16,7% en el 2019) comparado con los varones (39,9% en el 2007 a 22,1% en el 2019). Similares hallazgos se vieron en el tabaquismo activo (Tabla 2).

**Tabla 2:** Tendencia en el tiempo de la prevalencia de tabaquismo activo y pasivo según grupos de interés: Encuesta Mundial sobre Tabaquismo en Jóvenes 2007, 2014 y 2019.

Variables	Año de estudio			Valor de p ^a^
	2007	2014	2019	
	% (IC 95%)	% (IC 95%)	% (IC 95%)	
En mujeres				
Antecedente de tabaquismo previo	50,7 (46,9 - 54,5)	20,1 (16,1 - 24,0)	16,7 (13,4 - 19,9)	<0,001
Tabaquismo activo	21,2 (18,6 - 23,8)	6,5 (4,2 - 8,8)	4,4 (3,0 - 5,8)	<0,001
Tabaquismo pasivo en el hogar	24,6 (21,5 - 27,7)	12,9 (10,3 - 15,5)	10,8 (9,3 - 12,3)	<0,001
Tabaquismo pasivo diario en el hogar	4,0 (2,8 - 5,1)	1,6 (0,8 - 2,4)	1,5 (0,9 - 2,1)	<0.001
Tabaquismo pasivo fuera del hogar	47,0 (43,7 - 50,4)	40,5 (36,9 - 44,1)	39,2 (34,9 - 43,6)	0,007
Tabaquismo pasivo diario fuera del hogar	6,2 (5,0 - 7,5)	4,1 (2,9 - 5,3)	3,9 (3,0 - 4,8)	0,006
En varones				
Antecedente de tabaquismo previo	39,9 (36,7- 43,0)	30,6 (26,4 - 34,8)	22,1 (17,8 - 26,5)	<0,001
Tabaquismo activo	13,1 (10,5 - 15,7)	10,9 (7,6 - 14,1)	7,0 (5,2 - 8,8)	<0,001
Tabaquismo pasivo en el hogar	24,8 (21,5 - 28,1)	12,9 (10,6 - 15,1)	9,9 (7,6 - 12,3)	<0,001
Tabaquismo pasivo diario en el hogar	2,7 (1,8 - 3,5)	1,9 (1,0 - 2,9)	1,4 (0,7 - 2,1)	0,108
Tabaquismo pasivo fuera del hogar	45,7 (41,6 - 49,8)	38,3 (33,8 - 42,8)	33,4 (28,6 - 38,1)	<0,001
Tabaquismo pasivo diario fuera del hogar	5,6 (4,7 - 6,5)	5,9 (4,5 - 7,3)	4,2 (3,2 - 5,2)	0,093
En aquellos que nunca han fumado				
Tabaquismo pasivo en el hogar	20,1 (17,3 - 23,0)	8,2 (7,1 - 9,3)	7,7 (6,4 - 9,0)	<0,001
Tabaquismo pasivo diario en el hogar	2,1 (1,4 - 2,8)	1,0 (0,5 - 1,4)	0,7 (0,4 - 1,0)	<0,001
Tabaquismo pasivo fuera del hogar	38,0 (34,3 - 41,8)	32,5 (29,2 - 35,8)	31,0 (26,7 - 35,2)	0,026
Tabaquismo pasivo diario fuera del hogar	4,2 (3,4 - 5,0)	3,6 (2,8 - 4,3)	2,9 (2,0 - 3,7)	0,101

Todas las estimaciones fueron hechas tomando en cuenta el diseño del estudio.

a Valor de p calculado usando pruebas de tendencia tomando en cuenta el diseño de los estudios.

### Prevalencia y tendencia de tabaquismo pasivo

La prevalencia de tabaquismo pasivo en el hogar fue de 14,0% (IC 95%: 12,9% - 15,2%). Dicho estimado se redujo de 24,7% (IC 95%: 22,0% - 27,3%) en 2007 a 12,9% (IC 95%: 10,6% - 15,1%) en el 2014, para posteriormente caer a 10,4% (IC 95%: 9,0% - 11,7%) en el 2019 (p-valor de tendencias < 0,001). De otro lado, la prevalencia global de tabaquismo pasivo fuera del hogar fue de 39,4% (IC 95%: 37,2% - 41,6%), con una caída en los estimados de 46,3% (IC 95%: 43,7% - 49,0%) en el 2007 a 39,4% (IC 95%: 36,0% - 42,8%) en el 2014, y a 36,3% (IC 95%: 32,1% - 40,5%) en el 2019 (valor de p de tendencias < 0,001) (Figura 1A).

**Figura 1:** Tendencia en el tiempo de las prevalencias de (A) tabaquismo pasivo y (B) tabaquismo pasivo diario en adolescentes en Perú: Encuesta Mundial sobre Tabaquismo en Jóvenes 2007, 2014 y 2019.

En forma similar, la prevalencia de tabaquismo pasivo diario en el hogar fue de 1,9% (IC 95%: 1,5% - 2,3%), con una caída de 3,3% (IC 95%: 2,6% - 4,0%) en el 2007 a 1,8% (IC 95%: 1,1% - 2,5%) en el 2014, y luego a 1,5% (IC 95%: 0,9% - 2,0%) en el 2019 (p-valor de tendencias < 0,001). De otro lado, la prevalencia global de tabaquismo pasivo diario fuera del hogar fue de 4,7% (IC 95%: 4,2% - 5,3%), y similar a los anteriores estimados, hubo una caída en el tiempo desde 5,9% (IC 95%: 5,1% - 6,7%) en el 2007 a 5,0% (IC 95%: 4,0% - 5,9%) en el 2014, y luego a 4,0% (IC 95%: 3,3% - 4,8%) en el 2019 (valor de p de tendencias p = 0,007) (Figura 1B).

### Tabaquismo activo y pasivo según grupos de interés

La Tabla 2 muestra los estimados y tendencias de los diferentes marcadores de tabaquismo seleccionados de acuerdo a sexo y en aquellos sin antecedente de tabaquismo. En el caso de las mujeres, todos los indicadores cayeron a través del tiempo, pero no fue el caso para tabaquismo pasivo diario en el hogar (p-valor de tendencias = 0,108) y tabaquismo pasivo diario fuera del hogar (p-valor de tendencias = 0,093) en los varones.

En el grupo sin antecedente de tabaquismo previo (es decir, en aquellos que reportaron nunca haber fumado), todos los indicadores cayeron en el periodo de estudio; sin embargo, aunque la prevalencia de tabaquismo pasivo diario fuera del hogar cayó en dicho lapso, la diferencia fue no significativa (p-valor de tendencias = 0,101) (Tabla 2).

## DISCUSIÓN

De acuerdo con los resultados de nuestro análisis, desde el 2007 se ha observado una reducción en varios de los indicadores de tabaquismo en adolescentes peruanos, siendo mayor en mujeres que en varones. A pesar de que para el 2019, solo 1 de cada 5 adolescentes entre 12 y 16 años reportaron tener antecedente de tabaquismo, esto dista mucho de la meta de consumo de tabaco en menores de 18 años de edad. Asimismo, la prevalencia de tabaquismo pasivo, especialmente fuera del hogar, sigue siendo alta, lo cual puede ser más relevante en aquellos sin antecedente de tabaquismo previo.

El consumo activo de tabaco es un factor de riesgo prevenible de morbilidad y mortalidad en todo el mundo. A pesar de la reducción en el consumo de tabaco en el tiempo, la prevalencia de antecedente de tabaquismo previo y de consumo actual de tabaco (en los últimos 30 días) sigue siendo alta. Sin embargo, nuestros estimados de prevalencia del 2019 son más bajos que los de un análisis global sobre la prevalencia de consumo de tabaco en adolescentes entre 13 y 15 años ^5^: 6,1% en mujeres y 11,3% en varones (vs. 4,4% y 7,0%, respectivamente, en el presente trabajo), y más bajos que los de otros países de la región ^18^ ^,^ ^19^.

De forma similar, la exposición pasiva al tabaco es también prevenible y, a pesar de depender de la reducción del consumo activo, sus efectos son relevantes porque incrementa la probabilidad de convertir a un adolescente en fumador, así como incrementar la susceptibilidad al tabaquismo ^20^. La prevalencia de tabaquismo pasivo reportada aquí es mucho menor que la reportada en otros estudios a nivel global ^14^, tanto en el hogar como fuera del mismo ^15^. Sin embargo, la prevalencia de consumo de tabaco en forma pasiva sigue siendo alta, especialmente fuera del hogar.

El Perú firmó y ratificó el Convenio Marco para el Control del Tabaco (FCTC por sus siglas en inglés) desarrollado por la OMS en el 2004. Este marco incluye la prohibición total de la publicidad, promoción y patrocinio de los productos de tabaco; advertencias sanitarias fuertes en el paquete de cigarrillos; protección de la exposición al humo de tabaco en lugares de trabajo y públicos, así como en el transporte público, y medidas para reducir el comercio ilícito del tabaco ^21^. Estas iniciativas, y su posterior fortalecimiento, podrían explicar la reducción observada en los distintos indicadores.

Los cambios observados en la prevalencia de los distintos indicadores de tabaquismo en adolescentes sugieren que las políticas de control del tabaco han mejorado en el Perú, especialmente aquellas relacionadas con la protección contra el humo del tabaco en el hogar, en lugares públicos y en el transporte ^22^. Empero, nuestros resultados sugieren que se debe fortalecer las políticas sobre el uso de tabaco, especialmente fuera del hogar.

De acuerdo a un trabajo previo que usó información del GYTS del 2007 en Perú y lo comparó con otros países de la región, y a pesar de que se observa una reducción marcada en el uso de tabaco en adolescentes, Perú fue calificado por la OMS como uno de los países con más baja implementación de políticas antitabaco ^23^. Incluso en dicho reporte, Perú era el único país donde las ciudades tenían una alta exposición a tabaquismo pasivo. 

La promulgación de la Ley 29517 (2011) que prohibía el consumo de tabaco en lugares públicos en forma mucho más restrictiva que la ley anterior ^8^ puede haber ayudado en la subsecuente reducción a la exposición al tabaquismo pasivo. De esta forma, la protección de los adolescentes a las formas existentes de tabaquismo, especialmente la forma pasiva en el hogar y en lugares públicas, debería ser fundamental para reducir el inicio del consumo de tabaco en este grupo de edad ^24^. De acuerdo con nuestros resultados, es necesario, entonces, seguir fortaleciendo las políticas existentes para lograr una mayor reducción en los indicadores de tabaquismo activo y pasivo.

Este estudio utiliza los datos del GYTS en diferentes momentos en el Perú. Adicionalmente, utiliza muestras representativas con metodología y preguntas estandarizadas. Los resultados podrían tener implicaciones importantes en la generación y evaluación de las políticas para control del consumo de tabaco en adolescentes peruanos. Sin embargo, este estudio tiene algunas limitaciones que merecen discutirse. Primero, el GYTS usa un instrumento basado en autorreporte para determinar información sobre el consumo de tabaco y, por lo tanto, podría existir sesgos de memoria o de deseabilidad social que podrían afectar los resultados. Segundo, solo aquellos estudiantes que estuvieron presentes durante la realización de la encuesta fueron evaluados y, por ende, puede afectar la generalización de los resultados. Tercero, aunque las encuestas del 2014 y 2019 son de representatividad nacional, la del 2007 fue realizada solamente en algunas de las ciudades grandes del Peru (Huancayo, Ica, Lima, Trujillo y Tarapoto) ^25^, por lo tanto, mostrar una caída mayor de la que realmente podría haber sucedido. A pesar de esto, los resultados muestran una clara tendencia de reducción en los indicadores de tabaquismo obtenidos y es comparable con lo observado en otros estudios ^5^ ^,^ ^14^ ^,^ ^15^. Cuarto, los indicadores de tabaquismo pasivo fuera del hogar fueron evaluados usando una sola pregunta en la encuesta del 2007, y dos preguntas en las del 2014 y 2019, lo que podría afectar los resultados. Sin embargo, los estimados parecen ser consistentes con otros estudios ^23^. Finalmente, ciertas variables como el nivel socioeconómico no fueron colectadas como parte de la encuesta. Este factor podría ser de relevancia dado el costo elevado de los productos con tabaco, lo que podría ser útil para caracterizar mejor los resultados mostrados.

En conclusión, nuestros resultados muestran una reducción sostenida en varios de los indicadores de tabaquismo en adolescentes peruanos entre 12 y 16 años, siendo mayor en mujeres que en varones. Sin embargo, la prevalencia de tabaquismo pasivo fuera del hogar sigue siendo elevada, lo que podría requerir un fortalecimiento de las actuales políticas de control de tabaco en el país.
